# Ulcerative Unilateral Tinea Manuum Caused by Nannizzia gypsea

**DOI:** 10.7759/cureus.55576

**Published:** 2024-03-05

**Authors:** Jesús Iván Martínez-Ortega, Ilse Fernández-Reyna, Arely Gissell Ramirez Cibrian, Carlos Enrique Atoche Dieguez

**Affiliations:** 1 Dermatology, Instituto Dermatológico de Jalisco, Zapopan, MEX; 2 Mycology, Centro Dermatológico de Yucatan, Mérida, MEX; 3 General Practice, Universidad Autonoma de Campeche, Campeche, MEX; 4 Mycology, Centro Dermatológico de Yucatan, Yucatán, MEX

**Keywords:** yucatan peninsula, dermatophytes, nannizzia gypsea, ulcerative presentations, tinea manuum

## Abstract

Dermatophytes, fungi specialized in keratin degradation, are key agents in skin infections, commonly referred to as tineas. Tinea manuum, affecting the hands, typically presents in noninflammatory or inflammatory forms, with ulcerative manifestations rarely reported. *Nannizzia gypsea*, a relatively uncommon cause of tineas, exhibits variable prevalence influenced by geographic factors. This study investigates a case of Ulcerative Unilateral Tinea Manuum caused by *N. gypsea*, aiming to explore the differential diagnosis, pathogenesis, and management. A 23-year-old female from the Yucatan Peninsula presented with an ulcerated lesion on her left hand. Initially suspected as Leishmaniasis, subsequent examination revealed tinea manuum. The study discusses differential diagnoses, highlighting the rarity of ulcerative presentations in dermatophytosis, and explores potential pathogenic mechanisms. This case underscores the importance of considering dermatophytes in ulcerative skin lesions and suggests a comprehensive diagnostic approach, particularly in endemic regions.

## Introduction

Dermatophytes, specialized fungi with a predilection for keratin, play a pivotal role in causing skin infections, commonly known as tineas. When these infections affect the hands, they are termed tinea manuum. Generally, manifestations encompass two forms: noninflammatory, characterized by diffuse scaling and hyperkeratosis, and inflammatory, presenting as vesicular eruptions in a dyshidrotic or eczematoid form. However, there are limited reports documenting ulcerative presentations [1].

*Nannizzia gypsea*, a relatively uncommon cause of tineas, exhibits variable prevalence influenced by factors such as geography, with an approximate global incidence of 1% [2]. While there is no available data for the prevalence in the Yucatan Peninsula, preliminary findings from our single center revealed 3,706 cases of tinea, of which 97 (2.6%) were attributed to *N. gypsea*. Among these cases, 6% manifested as tinea mannum and a fraction presented as ulcers.

This study aims to academically explore the pursued differential diagnosis, shedding light on this rare presentation, delving into the underlying pathogenesis, and ultimately proposing a key takeaway recommendation.

## Case presentation

**Figure 1:**
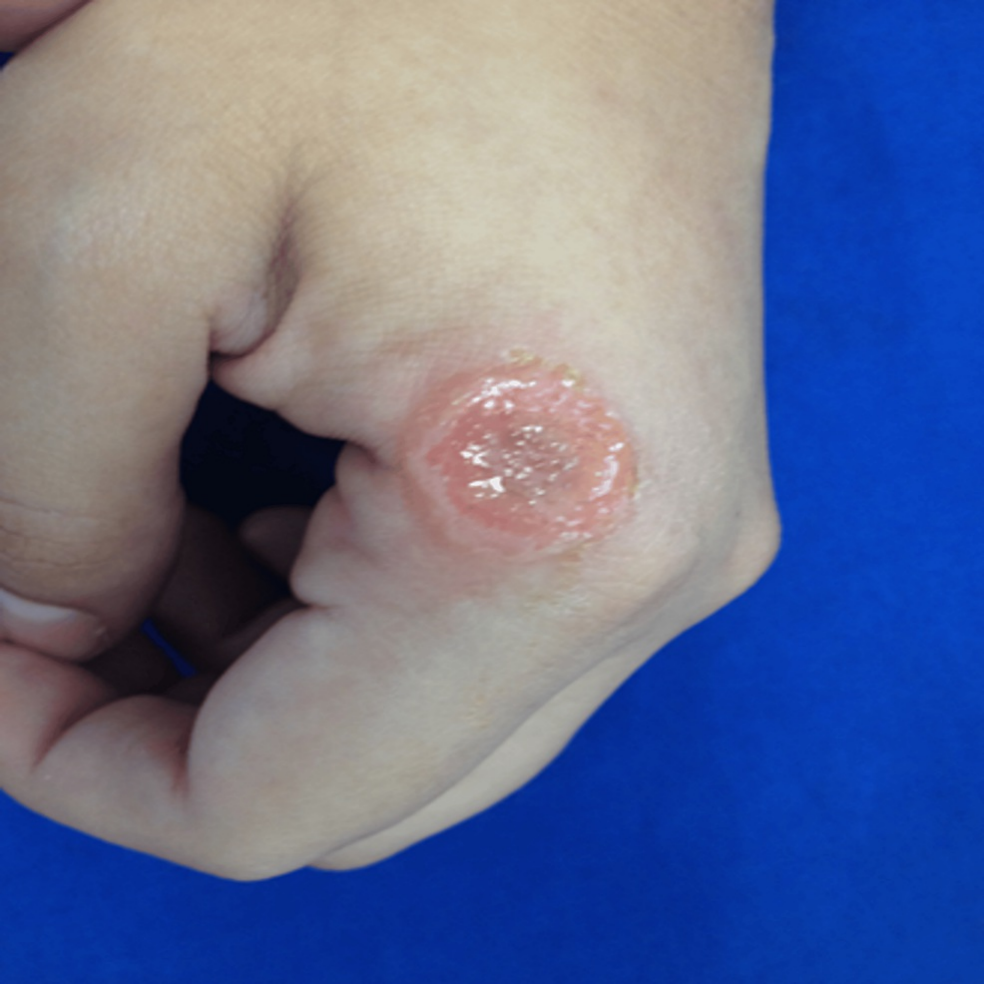
Ulcerative lesion on the dorsum of the left hand.

A 23-year-old female, native and residing in Yucatán, currently a student, presented at the dermatological clinic with a complaint of an ulcerated lesion on her left hand. The lesion exhibited elevated, well-defined, and scaly borders, accompanied by pruritus, and had evolved over one week (Figure 1).

The patient, with no significant medical history, denied the use of topical steroids or immunosuppressive medications. She mentioned owning a seemingly healthy cat for the past four years. Additionally, she had traveled to Tulum, Quintana Roo, for five days two weeks before the onset of the lesion, reporting multiple mosquito bites on various parts of her body during her stay.

**Figure 2:**
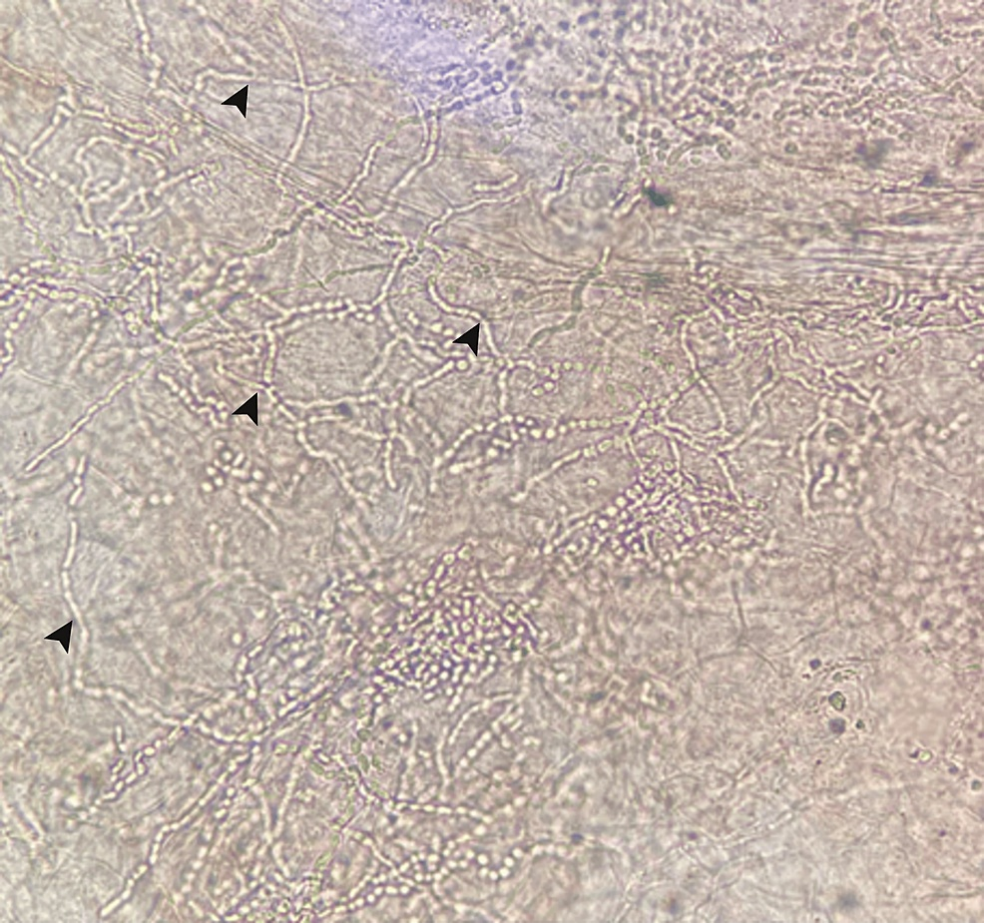
Direct examination with 20% KOH (x20). Abundant septate filaments (black arrowhead).

Initially suspected as leishmaniasis due to the recent trip to Quintana Roo, an endemic area, and mosquito bites, an imprint was taken, and the Wright stain was negative for leishmaniasis. Subsequent direct examination of the lesion with 20% KOH revealed abundant filaments, leading to the diagnosis of tinea manuum (Figure 2). Notably, there were no affections on other body sites. Treatment was initiated with 2% isoconazole cream applied topically every 12 hours, resulting in complete clinical resolution after two weeks.

**Figure 3:**
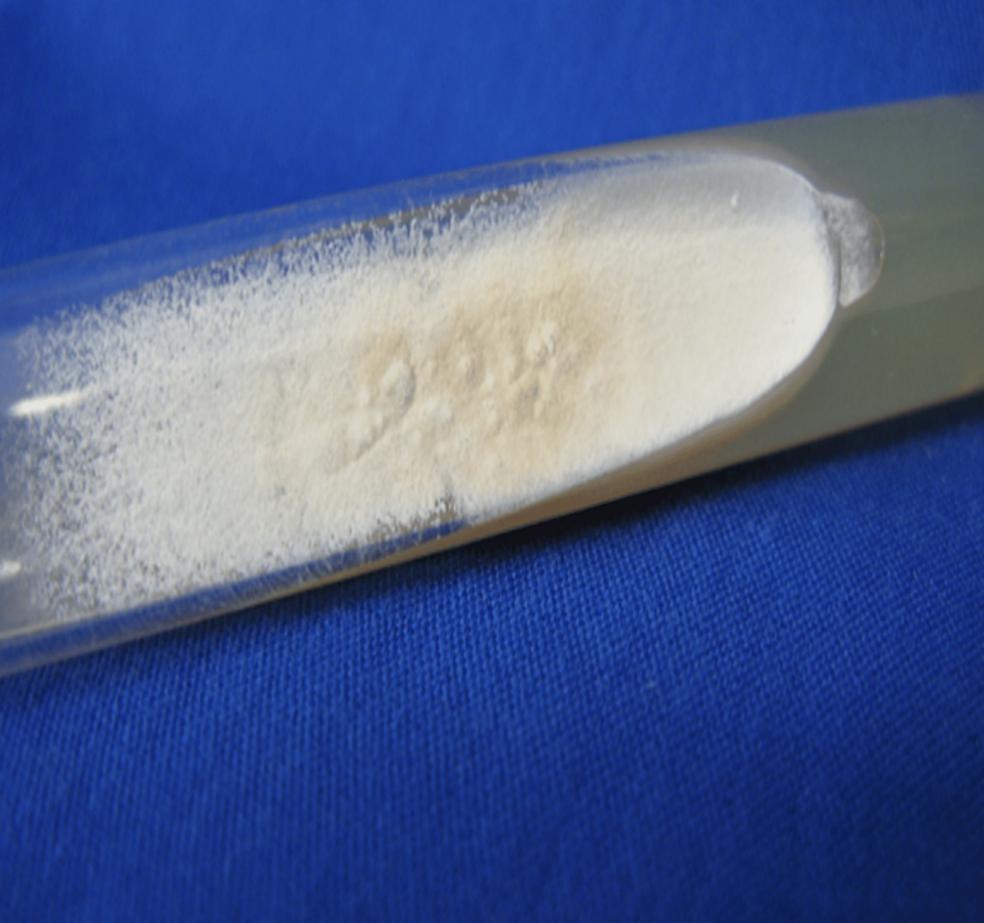
A Sabouraud agar culture. Macroscopic appearance at 10 days of Sabouraud agar colony, powdery and light beige. Image credit: Carlos Enrique Atoche Dieguez.

**Figure 4:**
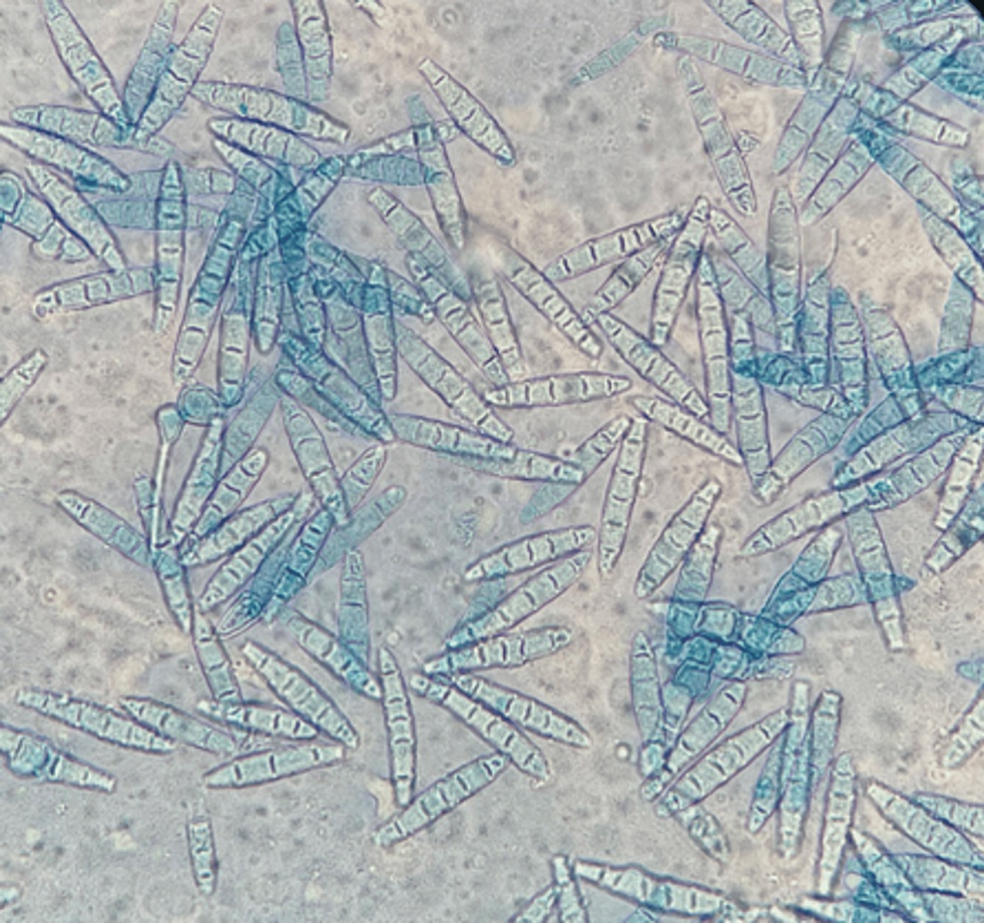
Microscopic examination of the culture with lactophenol blue (x20). Abundant macroconidia with thin walls, blunt edges, and internal septa.

A Sabouraud agar culture of the specimen had already grown at room temperature (29-34 °C) at this time, showing a powdery, beige-colored appearance (Figure 3). Microscopic examination with lactophenol blue revealed macro-aleuroconidia with thin walls and blunt edges, morphologically identifying the dermatophyte *N. gypsea* (Figure 4).

## Discussion

The Yucatan Peninsula, situated in the southeastern part of Mexico, is a sylvatic environment with a tropical climate favorable to the growth of *Leishmania*. It has recorded the highest number of leishmaniasis infections nationally, with the earliest worldwide record dating back to 1912 [3,4]. Therefore, when confronted with a chronic skin ulcer in our context, the first diagnosis to rule out is *Leishmania*. In different regions, initial suspicion should prioritize the prevalent local epidemiological infections. This includes diseases caused by other dimorphic fungi, such as sporotrichosis, blastomycosis, and coccidioidomycosis; certain parasitic infections like amoebas; bacterial infections, including ecthyma, as well as tuberculous and nontuberculous mycobacteriosis. It's crucial not to overlook other noninfectious conditions, ranging from neoplastic, like squamous cell carcinoma, to nonneoplastic etiologies, such as pyoderma gangrenosum and vasculitis. Additionally, miscellaneous causes like the recreational use of drugs (e.g., alkyl nitrites or *poppers*) should be considered.

The outermost epidermal layer, the stratum corneum, primarily consists of keratin. Dermatophytes, as they degrade keratin, are typically located superficially, often reaching up to the granulocyte stratum. Clinically, this manifests as round, scaly plaques with annular configurations following the advancement and proliferation of these fungi. Ulcers are rare, even in dermal dermatophytosis (when they reach deeper into the dermis through trauma), accounting for only a small percentage (5%) of deeper infections [5]. While an impaired epidermal barrier aggravated by patient scratching could be a straightforward and likely explanation, We contemplate two speculative possible scenarios. In the first, metalloproteinases unleashed by a highly inflammatory reaction to certain dermatophytes (geophilic and zoophilic) may cause an ulcer. Studies have shown that keratinocytes upregulate genes related to immune response when exposed to *N. gypsea*, whereas metabolic pathway genes are preferably expressed when exposed to *Trichophyton rubrum *[6]. The second scenario involves immunosuppressed patients, where a superimposed bacterial infection, such as *Staphylococcus aureus*, may introduce additional pathogenic pathways [7].* S. aureus*, a prevalent bacterium associated with skin infections, possesses over 20 exoenzymes, including exfoliative toxins A and B, hyaluronidase, lipases, and phospholipases. These bacterial enzymes, either alone or in synergy with fungal enzymes, may disrupt the epidermis down to the dermis [8].

While tinea incognito, by definition, simulates other skin disorders, a necessary criterion for its consideration is the use of topical and/or systemic immunosuppressants, primarily steroids. Despite thorough and targeted questioning, the patient consistently denied the use of any steroid-containing creams. As a result, the current case does not meet the criteria for categorizing it as Tinea incognito mimicking tropical ulcers [9].

Given that over 80% of patients with tinea manuum concurrently exhibit tinea pedis, manifestations may extend to one or both feet (termed two feet-one hand syndrome) or even involve both hands. Unilateral tinea mannum accounts for approximately 11% of cases [10].

## Conclusions

We propose considering the ulcerative presentation of tinea mannum as an inflammatory lesion, alongside the well-known and typical pustular and/or vesicular plaques on a highly erythematous base presentation. When encountered clinically, one should consider the possibility of a zoophilic/geophilic dermatophyte infection. Moreover, it is imperative to rule out local and systemic immunosuppressive factors, as well as superimposed bacterial infections, especially if a therapeutic antifungal response is absent during follow-up. Additionally, evaluating the involvement of nails, the other hand, and feet is essential for a comprehensive assessment. Further in-depth studies are essential to unravel the pathogenesis underlying ulcerative presentations in dermatophyte infections.
